# CPFTransformer: transformer fusion context pyramid medical image segmentation network

**DOI:** 10.3389/fnins.2023.1288366

**Published:** 2023-12-07

**Authors:** Jiao Li, Jinyu Ye, Ruixin Zhang, Yue Wu, Gebremedhin Samuel Berhane, Hongxia Deng, Hong Shi

**Affiliations:** ^1^College of Computer Science and Technology, Taiyuan University of Technology, Taiyuan, China; ^2^School of Artificial Intelligence, Shenzhen Polytechnic, Shenzhen, China

**Keywords:** medical image segmentation, Swin Transformer, Edge-Aware module, context pyramid fusion network, multiscale feature

## Abstract

**Introduction:**

The application of U-shaped convolutional neural network (CNN) methods in medical image segmentation tasks has yielded impressive results. However, this structure’s single-level context information extraction capability can lead to problems such as boundary blurring, so it needs to be improved. Additionally, the convolution operation’s inherent locality restricts its ability to capture global and long-distance semantic information interactions effectively. Conversely, the transformer model excels at capturing global information.

**Methods:**

Given these considerations, this paper presents a transformer fusion context pyramid medical image segmentation network (CPFTransformer). The CPFTransformer utilizes the Swin Transformer to integrate edge perception for segmentation edges. To effectively fuse global and multi-scale context information, we introduce an Edge-Aware module based on a context pyramid, which specifically emphasizes local features like edges and corners. Our approach employs a layered Swin Transformer with a shifted window mechanism as an encoder to extract contextual features. A decoder based on a symmetric Swin Transformer is employed for upsampling operations, thereby restoring the resolution of feature maps. The encoder and decoder are connected by an Edge-Aware module for the extraction of local features such as edges and corners.

**Results:**

Experimental evaluations on the Synapse multi-organ segmentation task and the ACDC dataset demonstrate the effectiveness of our method, yielding a segmentation accuracy of 79.87% (DSC) and 20.83% (HD) in the Synapse multi-organ segmentation task.

**Discussion:**

The method proposed in this paper, which combines the context pyramid mechanism and Transformer, enables fast and accurate automatic segmentation of medical images, thereby significantly enhancing the precision and reliability of medical diagnosis. Furthermore, the approach presented in this study can potentially be extended to image segmentation of other organs in the future.

## 1 Introduction

Automatic and accurate segmentation of medical images is of great significance to disease diagnosis. Image segmentation is an important part of medical image analysis, such as the segmentation of computed tomography (CT) images of chest organs and chambers in cardiac MRI images. Aided diagnosis and image-guided clinical surgery (Chen J. et al., 2021; Hatamizadeh et al., 2021), as well as accurate automatic segmentation, can be used to derive quantitative assessments of pathology or for subsequent diagnosis, treatment planning, and monitoring of disease progression.

Research methods about it emerge in an endless stream, and most of the state-of-the-art medical image segmentation frameworks are based on U-Net (Ronneberger et al., 2015) or its variants, using skip connections combined with encoder-decoder architecture; many algorithms follow this technical route. Usually, this structure preserves the single-granularity information of the encoder layer through skip connections. However, in doing so, it ignores the rich multi-scale spatial information, thus losing a lot of edge information, significantly affecting its performance in segmentation tasks. Furthermore, CNN’s inherent inductive bias makes it at a disadvantage to obtain global long-range semantic information interactions.

Therefore, it is difficult for CNN-based methods to learn global and long-range semantic information interactions. Recently, Transformer has also been applied to image processing (Carion et al., 2020), inspired by the great success of Transformer in natural language processing (Vaswani et al., 2017). Although CNN has achieved many excellent results in the field of image segmentation due to its characteristics of fast speed, low complexity, and high accuracy, it is not as good as Transformer in long-distance modeling. Therefore, the critical advantage of Transformer’s global attention combined with the excellent properties of CNN can build better segmentation networks. Currently, preliminary studies have attempted to apply Transformers to the field of medical image segmentation (Chen J. et al., 2021; Hatamizadeh et al., 2021; Cao et al., 2022). Now that both CNN and Transformer architectures demonstrate their unique advantages, it makes sense to combine the advantages of both architectures for a comprehensive analysis.

Specifically, the main contributions of this paper can be summarized as follows:

1. This paper presents a novel medical image segmentation network called the Transformer fusion context pyramid medical image segmentation network (CPFTransformer). To extract local features such as edges and corners, this paper designs an Edge-Aware (EA) block. This block uses convolution kernels of different sizes to extract features of different scales in multiple branches. High-level features express more semantic information, shallower features carry more detailed information. The features of the shallow backbone are mainly used to generate edge features, so it is best to extract local features such as edges and corners as much as possible to improve the segmentation effect.
2. To address the limitations of fixed-scale convolution operations in target segmentation, this paper uses Swin Transformer to construct a boundary-specific medical image segmentation network, maximizing the advantage of the Transformer’s focus on global information to build a symmetric encoder-decoder type architecture. In the encoder, self-attention is achieved locally to globally; in the decoder, global features are upsampled to the input resolution for the corresponding pixel-level segmentation prediction.
3. To enhance the precision and definition of segmented edges, this paper introduces a boundary loss and supplementary information to augment the region loss. The proposed approach incorporates a joint loss that combines the Dice loss, cross-entropy loss, and boundary loss, thereby improving the accuracy and clarity in segmentation tasks.
4. Experiments in the Synapse multi-organ CT image segmentation task and the ACDC MRI image segmentation task show that the Transformer fusion context pyramid medical image segmentation network achieves higher values in the Dice Coefficient and Hausdorff Distance evaluation metrics.

## 2 Related Work

In this section, topics related to medical image segmentation are discussed and reviewed in terms of encoder-decoder architecture, visual Transformer-based model, and contextual pyramidal feature fusion.

### 2.1 Encoder-decoder structure

Most state-of-the-art medical image segmentation frameworks are based on U-Net or its variants, which uses a skip-connected encoder-decoder architecture to extract semantic features through successive convolution and pooling operations. Many algorithms follow this technical route. Despite the simple network structure, it can be well applied to different medical segmentation tasks. 3DU-Net (Çiçek et al., 2016) replaces 2D with 3D convolution operations; Res-UNet (Xiao et al., 2018) replaces each sub-module of U-Net with residual connections respectively; and U-Net++ (Zhou et al., 2018) greatly reduces the number of parameters combines depth supervision. UNet3+ (Huang et al., 2020) proposed full-scale skip connections to use multi-scale information fully; R2U-Net (Alom et al., 2018) achieved better performance in different medical image segmentation tasks with the same computational load as U-Net; Ibtehaz and Rahman (2020) proposed MultiRes blocks to extract semantic information from multiple scales, and they also uses regular paths to alleviate the semantic gap between two symmetric encoder and decoder layers. Yuan et al. (2023) proposed CTC-Net, which designs two encoders by Swin Transformers and Residual CNNs to produce complementary features in Transformer and CNN domains, then uses a Cross-domain Fusion Block to blend them.

The common problem of these encoder-decoder-based architectures is that semantic features are usually extracted layer-by-layer during encoding, while the size and details of feature maps are recovered layer-by-layer during decoding. These approaches enable end-to-end pixel segmentation but lack rich contextual information, thus losing a lot of edge information. In medical image segmentation, it is often necessary to consider richer contextual information around the boundary region to be segmented.

### 2.2 Visual transformer and its variants

Previously, inspired by the great success of Transformers in natural language processing (Vaswani et al., 2017), researchers tried to introduce Transformer into the field of vision (Dosovitskiy et al., 2020). Vision Transformer (ViT), the first purely Transformer-based architecture, is proposed to perform image recognition task using 2D image patches with positional embeddings as input and performed on large datasets. When training, ViT performs on par with CNN-based methods. In 2021, Transformers-based methods such as (Zheng et al., 2021) were first applied to semantic segmentation. By modeling the global context of each layer of the Transformer, this encoder can be combined with a simple decoder to provide a powerful segmentation model. Furthermore, the Data Efficient Image Transformer (DeiT; Touvron et al., 2020) also shows that Transformer can be trained on medium-sized datasets. To ease the difficulty of training ViT, DeiT describes several training strategies to make ViT train well on ImageNet. Using the Swin Transformer as the visual backbone, Liu et al. (2021) achieved state-of-the-art performance in image classification, object detection, and semantic segmentation. Cao et al. proposed Swin-Unet (Cao et al., 2022) to leverage the power of the Swin Transformer for medical image segmentation. (Han et al., 2021; Liu et al., 2021; Valanarasu et al., 2021; Wang et al., 2021; Xie et al., 2021; Zhang et al., 2021, 2022; Guo et al., 2022) leverage features from Transformers and CNNs to improve segmentation models. Various combinations of Transformers and CNN are currently applied to multimodal brain tumor segmentation (Wang et al., 2021a) and 3D medical image segmentation (Peiris et al., 2021).

Common attention mechanisms usually focus on relationships in space and channels, capturing only local dependencies and failing to exploit the multi-scale contextual information around the target region fully. Therefore, multi-scale self-attention is crucial for capturing richer feature dependencies. There have been many attempts to apply Transformers to the field of medical image segmentation. However, little attention is paid to segmentation boundary information, and one of the key elements of segmentation is the segmentation edge.

### 2.3 The method of contextual feature pyramid

Multi-scale feature fusion has been extensively studied and proven effective for dense prediction tasks (Cai et al., 2016; Zhao et al., 2016; Chen Y. et al., 2017). The feature pyramid has the characteristics of different resolutions at different scales, and objects of different sizes can have appropriate feature representations at the corresponding scales. By fusing multi-scale information, objects of different sizes at different scales can be predicted, significantly improving the model’s performance.

There are roughly two ways to construct existing feature pyramids. The first is to generate layers of different resolutions through multiple downsampling, which is widely used. The more common applications are FPN (Lin et al., 2016) and YOLO_v3 (Redmon and Farhadi, 2018). FPN adopts the idea of divide and conquer, which means detecting large objects in the higher layers of the pyramid and small objects in the lower layers of the pyramid. The second comprises multiple branched convolutions with different void fractions and is currently used in ASPP, RFP, etc. Chen L. et al. (2018) proposed Atrous Spatial Pyramid Pooling (ASPP) to robustly segment objects by capturing image context at multiple scales. A simple approach is to resample the input image into a multi-resolution input pyramid, feed it to multiple or shared networks, and then aggregate the output features (Tompson et al., 2014; Chen L. et al., 2017; Chen C. et al., 2021). Feature pyramids fuse multi-scale features via pyramid pooling (Schlemper et al., 2019) or ASPP spatial pyramid pooling.

Convolutional neural network gathers information from neighboring pixels and loses spatial information due to pooling operations. Therefore, it is difficult for CNN to learn global and long-range semantic information interactions. Some studies have attempted to address this problem by using atrous convolutional layers, self-attention mechanisms (Zhao et al., 2016; Wang et al., 2017), and image pyramids. The multi-scale fusion based on the semantic map proposed in this paper is a global fusion of space and semantics, and different scale features at any location can help each other. Therefore, the network structure of the feature pyramid can handle the multi-scale variation problem in object detection with a slight increase in the amount of computation. It will be beneficial to apply it to the field of image segmentation.

## 3 Proposed method

**Figure 1:**
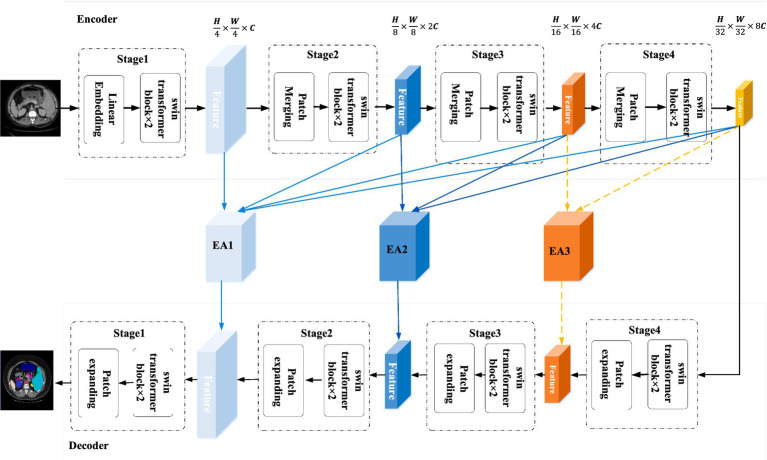
The structure of CPFTransformer, which consists of an encoder, two EA modules and a decoder. The encoder and decoder are constructed based on the Swin Transformer block. The EA module is composed of a multi-scale contextual pyramid module.

The general architecture of CPFTransformer proposed in this paper is shown in Figure 1. CPFTransformer consists of an encoder, a decoder, and two Edge-Aware modules. The reason there are three EA modules in Figure 1 is that EA modules can be theoretically added to these three positions. However, in section 4.5.2, it is proved that adding only the first two modules has the best effect. Therefore, in Figure 1, we mark the third module with a dotted line. The basic unit of the encoder and decoder is the Swin Transformer block (Liu et al., 2021).

First, the encoder segments the input medical image into 4 × 4 non-overlapping patch blocks and then projects the feature dimensions to arbitrary dimensions (denoted as C) through a linear embedding layer. The transformed token (patch token) is passed through a four-layer Swin Transformer block and a patch merging layer to generate hierarchical features. Specifically, the patch merging layer is responsible for downsampling and adding dimensionality, and the Swin Transformer block is responsible for feature learning. Inspired by U-Net, a symmetric decoder based on Swin Transformer is designed, which consists of Swin Transformer blocks and patch expanding layers. In contrast to the patch merging layer, the patch expanding layer is specifically designed to perform upsampling, it reshapes the feature maps of adjacent dimensions into an upsampled feature map with two times resolution. A final patch expanding layer is then used to perform 4× upsampling, recovering the image’s resolution and mapping the features to the input resolution size (W × H). Then a linear projection layer is used to output pixel-level segmentation predictions of these upsampled features.

In the middle of the encoder-decoder structure, instead of connecting with a simple jump connection, this paper introduces a kind of contextual pyramid module for connection, fusing the extracted contextual features with multi-scale features through the Edge-Aware module to compensate for the loss of spatial information at multiple scales, better extracting local features such as edges and corners (Sun et al., 2022), and providing the decoder with different levels of global contextual information by reconstructing the jump connection.

### 3.1 Swin Transformer block

The Swin Transformer block differs from the traditional Multiheaded Self-Attention (MSA) block in that it is constructed based on a shift window and comprises two consecutive Transformers. Each Swin Transformer block consists of a LayerNorm (LN) layer, a multiheaded self-attentive module, a residual connection, and a two-layer MLP with GELU nonlinearity. The two consecutive Transformer blocks employ a window-based multiheaded self-attentive (W-MSA) module and a shift-window-based multiheaded self-attentive (SW-MSA) module. Based on such a window division mechanism, the Swin Transformer block can be represented as:


(1) $${\hat{z}}^{l}=W‐MSA\left(LN\left(z^{l-1}\right)\right)+z^{l-1}$$

(2) $$z^{l}=MLP\left(LN\left({\hat{z}}^{l}\right)\right)+z^{l}$$

(3) $${\hat{z}}^{l+1}=SW‐MSA\left(LN\left(z^{l}\right)\right)+z^{l}$$

(4) $$z^{l+1}=MLP\left(LN\left({\hat{z}}^{l+1}\right)\right)+{\hat{z}}^{l+1}$$

where $z^{l}$ and MLP represent the output blocks of the (S)W-MSA module and the MLP, respectively.

Self-attention is calculated as follows:


(5) $$\mathrm{Attention}\left(Q,K,V\right)=\mathrm{SoftMax}\left(\frac{QK^{T}}{\sqrt{d}}+B\right)V$$

where $Q,K,V\in R^{M^{2}\times d}$ denotes the query, key, and value matrices. $M^{2}$ and d denote the number of patches in a window and the dimension of a query or key, respectively. Moreover, the values in B are taken from the bias matrix $\hat{B}\in R^{\left(2M-1\right)\times \left(2M+1\right)}$ .

### 3.2 Encoder

A medical image is partitioned into non-overlapping patches of size 4 × 4. First, C-dimensional tokens with a resolution of $\frac{H}{4}\times \frac{W}{4}$ are fed into two consecutive Swin Transformer blocks for representation learning, where the feature dimension and resolution are kept constant. By this partitioning method, the feature dimension of each patch is 4×4×3=48 . In addition, a linear embedding layer is used to project the feature dimension in an arbitrary dimension (denoted by C ). The transformed patches are passed through several Swin Transformer blocks and the patch merging layer to generate hierarchical feature representations. At the same time, the patch merging layer reduces the number of tokens (2× downsampling) and increases the feature dimension to two times the original one. This process will be repeated four times in the encoder. The patch merging layer is responsible for downsampling and dimensionality increase, and the Swin Transformer block is used for feature representation learning.

#### 3.2.1 Patch merging layer

The input patches are divided into four parts and connected by a patch merging layer. By doing this, the feature resolution is downsampled by a factor of 2. Also, since the concatenation operation results in a 4-fold increase in feature dimensionality, a linear layer is applied to the concatenated features to unify the feature dimensionality to two times the original dimensionality. The patch merging layer achieves the downsampling and feature dimension increase without convolution or interpolation operations.

### 3.3 Decoder

Corresponding to the encoder, a symmetric decoder is constructed, which is also built based on the Swin Transformer block. Therefore, corresponding to the patch merging layer used in the encoder, we build a patch expanding layer in the decoder for extracting depth features for upsampling. The patch expanding layer reshapes the feature map of the adjacent dimension into a higher-resolution feature map (2× upsampling). Accordingly, it reduces the feature dimension to half of the original dimension. Finally, a 4× upsampling is performed using the network structure’s last patch expanding layer to recover the image’s resolution features mapped to the input resolution, which is then passed through a linear projection layer for these upsampled features to output pixel-level segmentation predictions.

#### 3.3.1 Patch expanding layer

Using the first patch expanding layer as an example, a linear layer is applied to the input features $\left(\frac{W}{32}\times \frac{H}{32}\times 8C\right)$ to increase the feature dimension to twice the original dimension $\left(\frac{W}{32}\times \frac{H}{32}\times 16C\right)$ before upsampling. Then, we use a rearrangement operation to extend the resolution of the input features to twice the input resolution and reduce the feature size to one-fourth of the input size $\left(\frac{W}{32}\times \frac{H}{32}\times 16C\to \frac{W}{16}\times \frac{H}{16}\times 4C\right)$ . The patch expanding layer is the inverse operation of the patch merging layer. For the patch merging layer in encoder, in this paper, the patch expanding layer is specially designed in decoder for upsampling and feature dimension increase.

### 3.4 Edge-aware module

**Figure 2:**
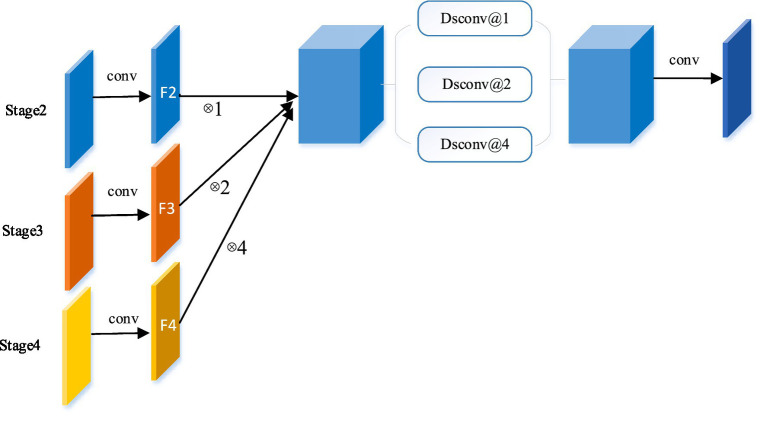
The Edge-Aware module. Taking the reconstructed skip connection at stage 2 as an example, by integrating the global context, the global information flow is transmitted from a higher stage (stage 3 and 4) to the decoder.

To address the limitations of fixed-scale convolutional operations in target segmentation, this paper designs an EA module to improve the robustness of the network structure through information at different scales. In addition, skip connections, commonly used in U-shaped networks, introduce irrelevant confusion and semantic gaps when the receiving fields do not match (Wang et al., 2022). In this paper, the EA module of the global context pyramid structure is proposed to solve these problems, as shown in Figure 2. In the EA module, the jump connections are reconstructed. And during the decoding process, the shallow features display detailed boundary information and also bring some background noise. Therefore, the EA module is used to extract edge features and further guide the decoder, while suppressing shallow noise and refining the contours of the object.

In this block, we use convolutional kernel operations of different sizes to extract features of different scales in multiple branches, enabling the network to learn more contextual information by fusing spatial information of different granularity. Based on the U-shaped structure, this paper first designs multiple EA modules between the encoder and decoder, aiming to provide the decoder with different levels of global contextual multi-scale information by reconfiguring the hopping connections. It is well known that higher-level features express more semantic information, while shallow-level features carry more details. Therefore, the features of shallow depth trunks are used to generate edge features. The proposed EA module extracts as many shallow features as possible for generating edge features.

In the EA module, the feature maps of this stage are combined with the feature maps of all higher stages to reconstruct the skip connections. Take the Stage 2 EA module for example, as shown in Figure 2, the features of each stage are first mapped to the same channel space as Stage 2 in a 3 × 3 convolution. In detail, features F3 and F4 are upsampled to the same size as F2 and connected. In order to extract global contextual information from different levels of feature maps, three separable convolutions (Chollet, 2016) (Dsconv@1, Dsconv@2, and Dsconv@4) with different dilation rates (1, 2, and 4) are used in parallel, where separable convolutions are used to reduce the model parameters. It is worth noting that the number rate of parallel paths and expansions varies with the number of fusion stages. Finally, the final feature map is obtained by convolution. The EA module can be summarized for each stage (regular convolution is ignored to simplify the formulation):


(6) $$G_{k}=C_{i=k}^{i=5}\left(D_{\mathrm{sconv}}@2^{i-k}\left(C_{i=k}^{i=5}\left(F_{k}⊗2^{i-k}\right)\right)\right)$$

where $G_{k}$ is the insertion of stage k, $F_{k}$ is the feature map of stage k in the encoder, $⊗2^{i-k}$ is the upsampling operation rate for $2^{i-k}$ , c denotes the concatenation operation and $D_{\mathrm{sconv}}@2^{i-k}$ is the separable dilation convolution expansion rate for $2^{i-k}$ .

To reduce the computational cost, the network in this paper uses only two EA modules. The global semantic high-level information flow can be gradually directed to different stages by introducing encoders and decoders between multiple EA modules.

### 3.5 Loss function

One of the main challenges in medical image segmentation is the imbalance of classification distribution. Traditional methods generally employ Dice loss or cross-entropy to perform the segmentation task. Loss functions widely used in convolutional neural network (CNN) segmentation, such as Dice loss or cross-entropy loss, which typically evaluate pixel-level accuracy, are based on the integration (summation) of the segmented region without global constraints on the segmentation shape. Models trained in this way often fail to produce complete segmentations with distribution shifts. For highly unbalanced segmentations, the values of these region losses vary significantly across segmentation classes, often by several orders of magnitude, which may affect training performance and stability. Moreover, the loss functions, such as BCE, IoU, and Dice, which are widely used in current segmentation tasks, do not penalize the incorrect segmentation of the boundaries.

Therefore, to further optimize the model in this paper, a boundary loss (Kervadec et al., 2021) is introduced in this paper, which appears as a distance metric on contour (or shape) space instead of regions. This alleviates the difficulty of region loss under highly unbalanced partitioning problems because it uses the integral over the region boundary (interface) rather than the unbalanced integral over the region. In addition, the boundary loss provides information that complements the region loss.

The expression of the boundary loss used in this paper is as follows:


(7) $$L_{B}\left(\theta \right)=\underset{\Omega }{\int }\phi_{G}\left(q\right)dq$$

where $s:\Omega \to \left\{0,1\right\}$ is binary indicator function of region $S:s\left(q\right)=1$ if q∈S belongs to the target and 0 otherwise. $\phi_{G}:\Omega \to R$ denotes the level set representation of boundary $\partial G:\phi_{G}\left(q\right)=-D_{G}\left(q\right)$ if q∈G and $\phi_{G}\left(q\right)=D_{G}\left(q\right)$ otherwise.

In the experiments, the joint loss $L_{\mathrm{total}}$ consisting of Dice loss, cross-entropy loss, and boundary loss will be used to perform all segmentation tasks in this paper.

## 4 Experiments

### 4.1 Datasets

#### 4.1.1 Synapse multi-organ segmentation dataset

The dataset includes 3,779 axial abdominal clinical CT images of 30 cases. 18 samples were divided into the training set and 12 samples into the test set. The volume of each CT image consists of 85 ~ 198 slices of 512 × 512 pixels, with a voxel spatial resolution of ([0.54 ~ 0.54] × [0.98 ~ 0.98] × [2.5 ~ 5.0]) $mm^{3}$ . In this paper, the average Dice similarity coefficient (DSC) and average Hausdorff distance (HD) on eight abdominal organs (aorta, gallbladder, spleen, left kidney, right kidney, liver, pancreas, and stomach) were used as evaluation indexes.

#### 4.1.2 ACDC dataset

ACDC is a public cardiac MRI dataset that includes a sample of 100 cases. A series of short-axis slices covering the heart from the base of the left ventricle to the apex with a thickness of 5–8 mm. The spatial resolution in the short-axis plane ranges from 0.83 to 1.75 $mm^{2}/\mathrm{pixel}$ , corresponding to labels including left ventricle (LV), right ventricle (RV), and myocardium (MYO). The dataset was divided into 70 training samples (1,930 axial slices), 10 validation samples, and 20 test samples.

### 4.2 Experimental setup

#### 4.2.1 Environment

CPFTransformer is implemented based on Python 3.6 and Pytorch 1.7.0. For all training cases, data enhancements such as flips and rotations are used to increase the diversity of the data. The input image size is set to 224 × 224, and the patch size is set to 4. The model in this paper is trained on an Nvidia V100 GPU with 32 GB of memory.

During the training period, the batch size is 24 and an SGD optimizer with a momentum of 0.9 and a weight decay of 1e−4 is used to optimize the back propagation model in this paper.

#### 4.2.2 Evaluation metrics

Two types of metrics are used in this paper to evaluate all models.

Dice similarity coefficient (DSC) for evaluating the degree of overlap between prediction and ground truth segmentation map:


(8) $$DSC=\frac{2\left|P\cap G\right|}{\left|P\right|+\left|G\right|}$$

where P is the predicted segmentation map and G is the ground truth.

Hausdorff distance (HD), which measures the maximum symmetric distance between two segmentation maps:


(9) $$d_{H}\left(P,G\right)=\max\left\{\underset{p\in P}{\sup}\underset{g\in G}{\inf}d\left(p,g\right),\underset{g\in G}{\sup}\underset{p\in P}{\inf}d\left(p,g\right)\right\}$$

where $d\left(\text{.}\right)$ is the Euclidean distance, sup and inf are the upper and lower extremes, respectively. In this paper, 95% HD is used to eliminate the effect of a minimal subset of outliers.

### 4.3 Experiment results on synapse dataset

On the Synapse multi-organ CT dataset, a comparison of the CPFTransformer proposed in this paper with previous state-of-the-art methods is shown in Table 1. The experimental results show that the segmentation accuracy of the Swin Transformer fused multi-scale contextual pyramid-based method in this paper achieves 79.87% (DSC ↑ ) and 20.83% (HD ↓ ). Compared with Unet and the recent TransUnet, Swinunet, and MTunet (Wang et al., 2021) methods, the algorithm in this paper has a slight improvement in the DSC evaluation metric and HD evaluation metric, which indicates that the method in this paper can achieve better segmentation edge prediction.

**Table 1:** Quantitative comparison of synapse dataset using the most advanced algorithm.

Method	DSC (%)	HD (mm)	Aorta	Gallbladder	Kidney (L)	Kidney (R)	Liver	Pancreas	Spleen	Stomach
U-net	76.85	39.70	89.07	**69.72**	77.77	68.60	93.43	53.98	86.67	75.58
R50U-Net	74.68	36.87	84.18	62.84	79.19	71.29	93.35	48.23	84.41	73.92
Att-UNet	77.77	36.02	**89.55**	68.88	77.98	71.11	93.57	58.04	87.30	75.75
Transunet	77.48	31.69	87.23	63.13	81.87	77.02	94.08	55.86	85.08	75.62
MTunet	78.59	26.59	87.92	64.99	81.47	77.29	93.06	**59.46**	87.75	76.81
Swinunet	79.13	21.55	85.47	66.53	**83.28**	**79.61**	94.29	56.58	**90.66**	76.60
CPFTrans-former	**79.87**	**20.83**	87.71	68.78	83.19	79.15	**94.37**	58.47	90.35	**76.93**

Columns 4–11 represent the Deice similarity coefficients obtained on each organ. The best values are bolded.

**Figure 3:**
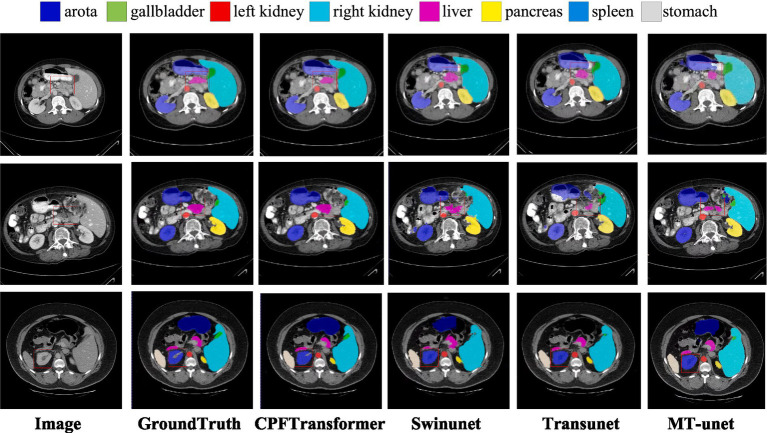
Visual comparison of segmentation results of different methods in Synapse dataset. The first column is the original image, and the second column is the ground truth. The area highlighted by the red box shows that CPFTransformer performs better segmentation than other most advanced methods and results, and the segmentation edge is closer to the basic facts.

The segmentation results of different methods on the Synapse multi-organ CT dataset are shown in Figure 3. The figure shows that the CNN-based methods tend to have over-segmentation problems, which may be due to the local nature of convolutional operations. This paper demonstrates a medical image segmentation network based on Swin Transformer’s contextual pyramid fusion multi-scale feature combination. The network captures rich multi-scale contextual information using pyramid structure fusion. It computes the convolution of local correlation between adjacent pixels, which performs well in extracting local features such as edges and corners to obtain better segmentation results.

Table 1 provides a quantitative evaluation of the experimental results. U-net is the original method for generating adversarial networks; Transunet uses the encoding structure of Transformer’s generative model and the decoding structure of CNN; Swinunet is the codec structure using the pure Transformer method; CPFTransformer is the method proposed in this paper. The table shows that using the contextual pyramid structure combined with the Swin Transformer method, DSC is improved to 79.87%, and HD is improved to 20.83%. The experiments demonstrate that introducing the contextual pyramid mechanism in the Swin Transformer network can effectively and precisely target the edges to accomplish the task of abdominal multi-organ segmentation.

**Figure 4:**
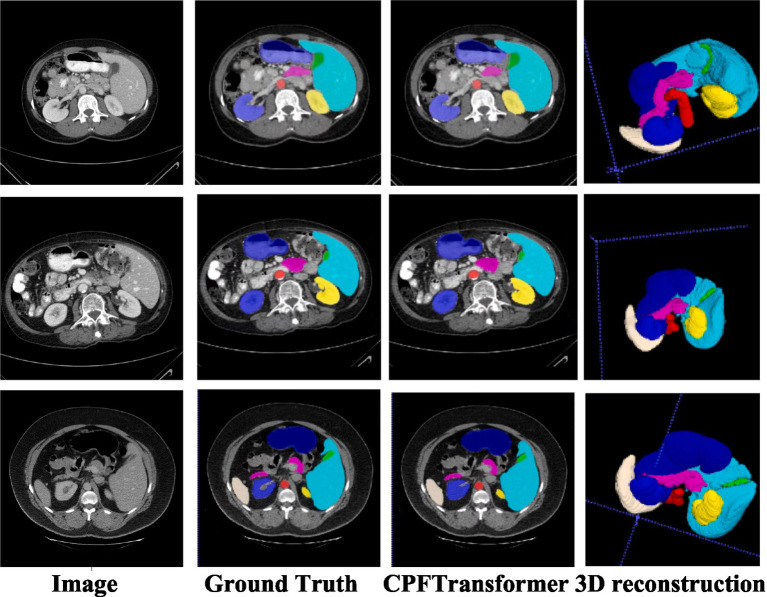
Synapse segmentation results after 3D reconstruction.

To verify the performance of the model, it was compared with an expert segmentation approach, i.e., labeling. Then the pre-processed slicing results were reduced to the nii format dataset by 3D reconstruction to compare the segmentation results under 3D data. Figure 4 shows the segmentation results of the model under different layers of the Synapse dataset. The first column is the original image after slicing the Synapse dataset, the second column is the labeling result, the third column is the segmentation result of the network model proposed in this paper, and the fourth column is the segmentation result of the 3D reconstruction. As can be seen from the figure, by comparing with the results of expert segmentation in the second column, the experimental segmentation results of this paper in the third column are segmented precisely in detail and similar to the labeled segmentation results. Moreover, the edges are rounded after the 3D reconstruction of the sliced data.

### 4.4 Experiment results On ACDC dataset

**Figure 5:**
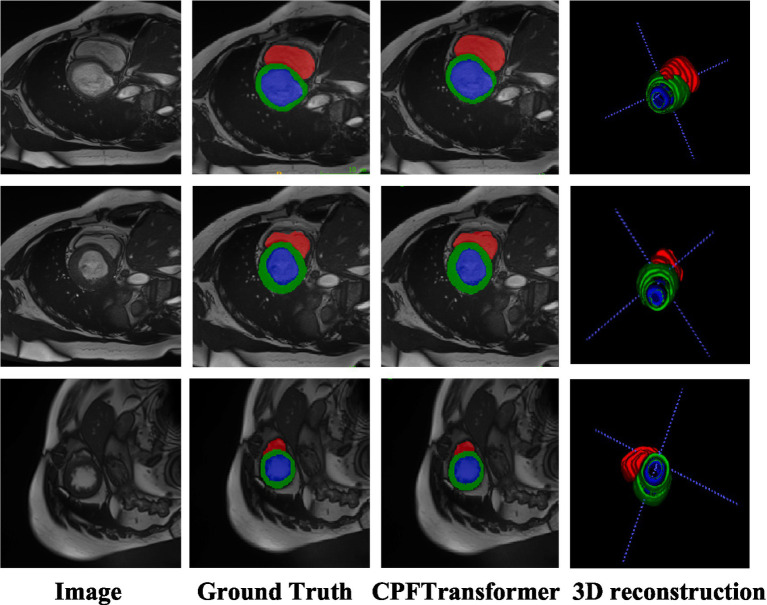
ACDC segmentation results and segmentation results after 3D reconstruction.

Like the Synapse dataset, the proposed CPFTransformer is in the ACDC dataset to perform medical image segmentation. The experimental results are summarized in Table 2. Using MRI pattern image data as input, CPFTransformer can still achieve excellent performance with an accuracy of 90.00%. This indicates that our method has good generalization ability and robustness. As shown in Figure 5, the first column is the original image after slicing the ACDC dataset; the second column is the labeling result, the third column is the segmentation result of the network model proposed in this paper, and the fourth column is the segmentation result of 3D reconstruction. As can be seen from the figure, by comparing the results with those of the labeled results in the second column, the experimental segmentation results of this paper in the third column are segmented accurately in detail, basically similar to the labeled segmentation results. The edge rounding can be seen after the 3D reconstruction of the sliced data.

**Table 2:** Quantitative comparison of ACDC dataset using the most advanced algorithm.

Method	DSC (%)	RV	Myo	LV
R50AttnUNet (Chen C. et al., 2021)	86.90	83.27	84.33	93.53
R50 ViT (Wang et al., 2021b)	86.19	82.51	83.01	93.05
TransUnet (Chen J. et al., 2021)	89.71	86.67	87.27	95.18
SwinUnet (Zheng et al., 2021)	88.07	85.77	84.42	94.03
CPFTransformer	**91.36**	**88.76**	**90.01**	**96.06**

Columns 3–5 represent the Deice similarity coefficients obtained on each organ. The best values are bolded.

### 4.5 Ablation experiment

To investigate the effect of different factors on the model performance, an ablation study was conducted on the Synapse dataset in this paper. Same as training, 18 samples are used for training and 12 samples are used for testing.

#### 4.5.1 The effect of position of the EA module

**Figure 6:**
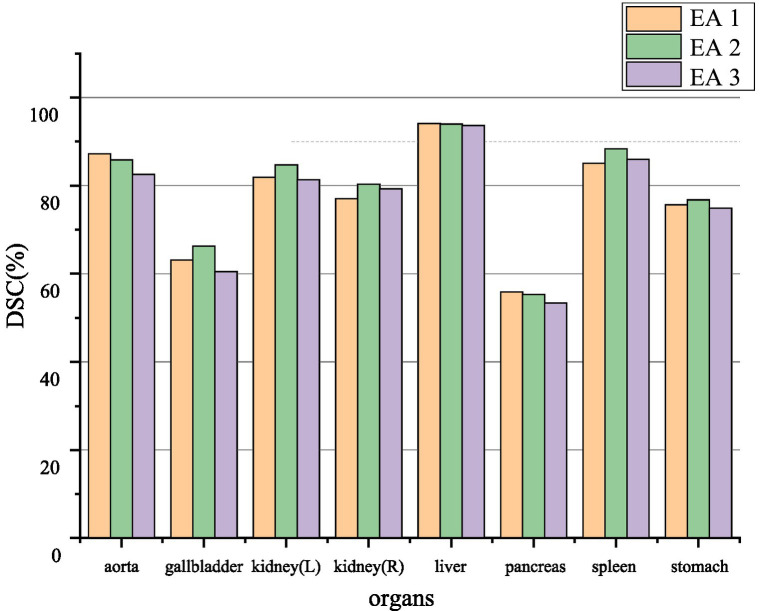
Ablation experiment of synapse dataset. EA1, EA2, and EA3, respectively, represent the effect between different positions where the edge sensing module is located as stage 1, stage 2, and stage 3.

The EA module in this paper was added to the 1/4, 1/8, and 1/16, 1/32 resolution scales to explore the effect of position on segmentation performance. The segmentation performance of the model can be seen in Figure 6. Positions 1 and 2 generally perform more accurately than position three. Therefore, to make the model more robust, the case of position three will not be used in this paper.

#### 4.5.2 The effect of number of the EA module

The discussion of the number of EA modules in this paper incorporates location restrictions so that there can be a maximum of three blocks and a minimum of zero blocks. The effect of the number on the segmentation performance is explored. The segmentation performance of the model can be seen quantitatively in Table 3. With three EA modules causing redundancy and mediocre results, the effect of two blocks is generally better than the one block. Therefore, two EA modules will be used in this paper to make the model more robust.

**Table 3:** Ablation experiment of synapse dataset.

Number of modules	DSC (%)	HD (mm)
Three (EA1, 2, 3)	76.34	25.37
Two (EA1, 2)	**79.87**	**20.53**
Two (EA1, 3)	78.82	23.24
Two (EA2, 3)	77.65	23.07
One (EA1)	76.34	25.37
One (EA2)	77.37	23.53
One (EA3)	75.82	23.24
Zero	79.13	21.55

The effect of application and network between different EA modules and different locations. The best values are bolded.

In this paper, through ablation experiments, we conclude about the effect of the number of EA modules. Adding three EA modules causes too much redundancy in the extracted information, resulting in poor results, and segmenting the edges of low-dimensional information features is more beneficial. When adding EA1 and EA2 modules in the low-dimensional features, the effect is better, and the high-resolution low-level features and low-resolution high-level features are fused to help delineate the detailed boundary.

#### 4.5.3 Effect of loss function

The loss functions used in this paper include Dice loss and cross-entropy loss commonly used in segmentation and boundary loss is introduced. After performing ablation experiments on the three losses, as shown in Table 4, it is found that in order to make the segmentation effect better, this paper will adopt a joint training method for the three losses.

**Table 4:** Ablation experiment of synapse dataset.

Loss	DSC (%)	HD (mm)
Lce	76.47	29.25
Ldice	75.23	31.39
Lce + Ldice	78.96	21.32
Lce + Lb	77.98	26.93
Ldice + Lb	76.70	25.37
Lce + Ldice + Lb	**79.87**	**20.53**

Different effects of loss function application and network. The best values are bolded.

#### 4.5.4 Discussion

Although CPFTransformer can achieve good results in medical image segmentation, it also has a significant disadvantage. Compared with the traditional convolutional medical image segmentation models, the Transformer model combined with the edge-aware module of the context pyramid, although it integrates more global multi-scale context information, it also leads to a slower overall convergence speed and training. Time becomes longer.

There are two main directions for the future of this research. First, we will explore compressing CPFTransformer to eliminate redundant parameters and reduce computational overhead while maintaining its effectiveness. Finally, CPFTransformer is designed based on 2D images, but 3D medical images have essential application value. In the future, CPFTransformer will be further improved to make it suitable for 3D medical image segmentation tasks.

## 5 Conclusion

This paper proposes an edge-aware medical image segmentation network (CPFTransformer) using Swin Transformer. We design an Edge-Aware module based on a context pyramid to fuse global multi-scale context information, mainly for local features such as corners. It focuses on addressing the weakness of global multi-scale contextual information capture and integration in U-shaped networks, and a novel Edge-Aware module is inserted into the U-shaped framework with a context-pyramid-based boundary-aware module to develop and fuse rich global multi-scale context information. Fusing high-resolution low-level features and low-resolution high-level features helps delineate detailed segmentation edges.

This paper conducts comprehensive experiments on different types of medical image segmentation tasks to verify the effectiveness and generality of the proposed CPFTransformer, including the abdominal multi-organ segmentation task and ACDC dataset. Experiments on segmentation tasks show that the edge-aware context pyramid network based on Swin Transformer performs better. Our proposed CPFTransformer achieves excellent and consistent performance on two different segmentation tasks, which indicates that the proposed CPFTransformer is more practical and scalable than the others. Our method can achieve better performance with further processing and can be extended to other medical image segmentation tasks, which is our recent work.

## Data availability statement

Publicly available datasets were analyzed in this study. The data are publicly available at https://www.synapse.org (Synapse) and https://acdc.creatis.insa-lyon.fr (ACDC).

## Author contributions

JL: Conceptualization, Methodology, Project administration, Software, Writing – original draft, Writing – review & editing. JY: Project administration, Validation, Writing – original draft. RZ: Supervision, Writing – review & editing. YW: Supervision, Writing – review & editing. GB: Supervision, Writing – review & editing. HD: Formal analysis, Funding acquisition, Resources, Supervision, Writing – review & editing. HS: Project administration, Validation, Writing – review & editing.
